# *IL28B* Genetic Variations in Patients with Recurrent Herpes Simplex Keratitis

**DOI:** 10.3390/medicina55100642

**Published:** 2019-09-26

**Authors:** Savić Borivoje, Stanojlović Svetlana, Hadži-Milić Milan, Đonović Nela, Milošević-Đorđević Olivera, Milisavljević Filip, Stojković Milenko, Pajić Srbislav

**Affiliations:** 1 Clinic for Eye Diseases, Clinical Center of Serbia, 11000 Belgrade, Serbia; sb.bora@gmail.com (S.B.); stanojlovic.svetlana@gmail.com (S.S.); milenko.stojkovic@mfub.bg.ac.rs (S.M.); 2 Faculty of Medicine, University of Belgrade, 11000 Belgrade, Serbia; 3 Department of Surgery, Orthopedics and Ophthalmology, Faculty of Veterinary Medicine, 11000 Belgrade, Serbia; milanhmilic@gmail.com; 4 Department of Hygiene and Ecology, Faculty of Medical Sciences, University of Kragujevac, 34000 Kragujevac, Serbia; nela@medf.kg.ac.rs; 5 Department of Genetics, Faculty of Medical Science, University of Kragujevac, 34000 Kragujevac, Serbia; olivera@kg.ac.rs; 6 Department of Biology and Ecology, Faculty of Science, University of Kragujevac, 34000 Kragujevac, Serbia; 7 Clinic of Neurosurgery, Clinical Center of Serbia, 11000 Belgrade, Serbia; milisavljevic93@gmail.com; 8 Emergency Center, Clinic for Emergency surgery, Clinical Center of Serbia, 11000 Belgrade, Serbia

**Keywords:** type III interferons (*IL28B*) genotype, keratitis, herpes simplex virus type 1 (HSV-1)

## Abstract

*Background and objectives*: Recurrent herpes simplex keratitis (HSK) is the most common cause of corneal blindness in the developed world. A relationship between host gene polymorphisms and the recurrence of herpes simplex virus (HSV) infection has previously been proposed. Thus, the aim of this study was to investigate a potential association between the *IL28B* host genotype and recurrent HSK. *Materials and Methods*: Eighty patients older than 18 years of age of both genders with a history of recurrent herpes simplex labialis (HSL) were considered for inclusion. Seventy-five of these patients were found to be seropositive for HSV-1 and were subsequently enrolled in the study. Twenty-four of the enrolled patients also had a history of recurrent HSK associated with severe corneal scarring and visual acuity deterioration. Total DNA was isolated from whole blood samples. A single-nucleotide polymorphism (SNP) rs12979860 near the *IL28B* gene on chromosome 19 was genotyped. *Results*: A significant association was observed between recurrent HSK and two SNPs of the *IL28B* genotype (CCrs12979860 and CTrs12979860, *p* < 0.01). The variation CCrs12979860 showed a significantly greater association with HSK (16 out of 26 patients) compared with CTrs12979860 (8 out of 34 patients). *Conclusion*: Seropositive individuals with a history of recurrent HSK are likely to have the CC *IL28B* genotype. This genotype may be related to incomplete control of the infection and more frequent periodical viral shedding along the first nerve branch of the trigeminal ganglion, which clinically manifests as recurrent herpes keratitis. The clinical manifestation of recurrent HSV-1 infection seems to be influenced by polymorphism of the *IL28B* genotype.

## 1. Introduction

Herpes simplex virus (HSV) infection is ubiquitous in humans, infecting 50–90% of the world’s population [1,2]. HSV-1 causes a wide range of diseases, including herpes labialis, gingivostomatitis, and keratitis. The prevalence of ocular HSV disease has been estimated to be 149 in a population of 100,000 and in about one-fifth of the cases, leads to the development of stromal herpetic keratitis [3]. Herpes simplex keratitis (HSK) is one of the leading causes of corneal blindness, primarily because of its recurrent nature [4]. 

After primary infection of the sensory nerves innervating the skin and mucosal epithelium, HSV-1 has the ability to establish lifelong latency, most commonly within the trigeminal ganglion. The HSV-1 virus uses actin and microtubules for retrograde transport from the plasma membrane during entry and exit along axons. The composition of the viral particles, especially their protein complement, seems to determine the direction of transport during the entry and exit of the virus in neuronal and non-neuronal cells [5]. It has been estimated that one-third of the world’s population suffers from recurrent HSV infections [3]. Upon periodical reactivation, the virus may follow any of the three branches of the fifth cranial nerve, regardless of the innervation branch of the primary HSV infection area. It is believed that cycles of HSV reactivation in latently infected neurons accompanied by anterograde axonal spread to the cornea lead to recurrent infections and scarring of the cornea [6]. Antiviral cytokine secretion as a result of host immunity plays a vital role in virus clearance; however, the concurrent inflammatory response is a major cause of corneal scarring, leading to vision loss [2,3,7]. 

The production of Type I interferons (IFNs) is most important for controlling viral replication [8]. Accordingly, enhanced HSV virulence has been reported in Type I IFN-receptor-deficient mice [9]. Interleukin (IL) 28B (interferon (IFN)-λ3), together with IL-28A and IL-29 (also termed IFN-λ1 and IFN-λ2) constitute a new subfamily within the IL-10 interferon family [10]. Indeed, IFN-λ exhibits a number of biological characteristics that are similar to those of IFN-α/β, including antiviral activity, antiproliferative activity, and in vivo antitumor activity. By signaling through the heterodimeric IL-28Rα/IL–10Rβ complex, IFN-λ performs antiviral and immunoregulatory activities [11,12]. It has also been shown that IFN-λ-mediated antiviral activity is linked to the activation of type I IFN-stimulated gene factor 3 (ISGF3) and the induction of IFN-α/β- and IFN-λ-stimulated antiviral genes [13]. This provides a potential mechanism for the inhibitory effect of IFN-λ on HSV-1. Additionally, IFN-λ has the ability to activate the same JAK-STAT (Janus kinase-signal transducer and activator transcription factor) and MAP (mitogen-activated protein) kinase pathway as Type I IFNs [14,15,16].

Recent studies have reported a relationship between a specific polymorphism of *IL28B* (rs12979860) and HSV-1 reactivation in patients with herpes labialis [17], suggesting that polymorphisms in genes involved in antiviral responses might modulate the risk of developing recurrent HSV infection. Of note, this polymorphism was also reported to have a positive correlation with IFN-λ3 serum levels and the treatment outcome in patients with hepatitis C virus infection [18,19]. Treatment with IFN-λ also inhibited virus replication and inflammatory reaction in an experimental model of HSK in mice [20].

The aim of this study was to evaluate possible correlations between host polymorphisms of the *IL28B* genotype and recurrent HSV keratitis in HSV-1 IgG-seropositive patients. To the best of our knowledge, the potential relationship between *IL28B* genotype and recurrent HSK has not previously been previously reported.

## 2. Materials and Methods

### 2.1. Study Setting

This was an in vitro experimental study using material of human origin. The study was conducted in accordance with the Institutional Review Board regulations, adhered to the tenets of the Declaration of Helsinki, and informed consent was obtained from all participants.

The sample consisted of 80 patients over the age of 18 of both genders. All participants filled out a questionnaire to confirm their history of recurrent herpes simplex labialis (HSL) infection (cold sores). In addition, blood samples were collected from all patients in order to confirm the HSV1 IgG-positive status. Seventy-five of the tested patients were seropositive for HSV-1 and were subsequently enrolled in the study.

Twenty-four of the enrolled patients also had a history of recurrent HSV keratitis, leading to severe corneal scarring and, consequently, visual acuity deterioration. Recurrences were classified as epithelial keratitis, stromal keratitis, endothelitis, iridocyclitis, or combinations of these conditions. All patients were followed up for at least one year, from January to December 2018, at the Clinic for Eye Diseases, Clinical Centre of Serbia, Belgrade. Participants with no history of herpetic eye disease were voluntary employees from the Scientific Institute of Veterinary Medicine of Serbia. Genotyping for *IL28B* (rs12979860snp) was performed in the laboratory of the Scientific Institute of Veterinary Medicine of Serbia.

### 2.2. Inclusion and Exclusion Criteria

The study included participants with a history of recurrent HSL infection and confirmed HSV-1 IgG-seropositive status. Participants lacking anti-HSV-1 IgG antibodies were excluded (5 out of 80). This selection step resulted in a main study sample of 75 subjects.

Inclusion criteria for patients exhibiting recurrent herpetic eye disease were as follows: recurrent herpetic keratitis associated with corneal scarring and neovascularization and significant deterioration of visual acuity (less than 6/60, Snellen).

Exclusion criteria were as follows: a history of associated ocular comorbidities and previous ocular surgery, systemic and neurological diseases.

### 2.3. Immunological Analyses

Five milliliters of peripheral venous blood was collected from each patient in order to identify HSV-1 IgG status. An enzyme immunoassay was conducted for the qualitative determination of IgG class antibodies against HSV Type 1+2 in human serum or plasma (GenWay Biotech, Inc., San Diego, CA, USA). The cut-off value was HSV1 IgG < 9NTU.

### 2.4. Determination of IL28B Genotype

Five milliliters of peripheral venous blood was collected from each patient in order to identify the polymorphic gene *IL28B* rs12979860.

Total DNA was isolated using a commercial QIAamp DNA Blood mini kit (QIAGEN, Hilden, Germany) in accordance with the manufacturer’s instructions. The single-nucleotide polymorphism (SNP) rs12979860, near the *IL28B* gene on chromosome 19, was genotyped.

**Figure 1:**
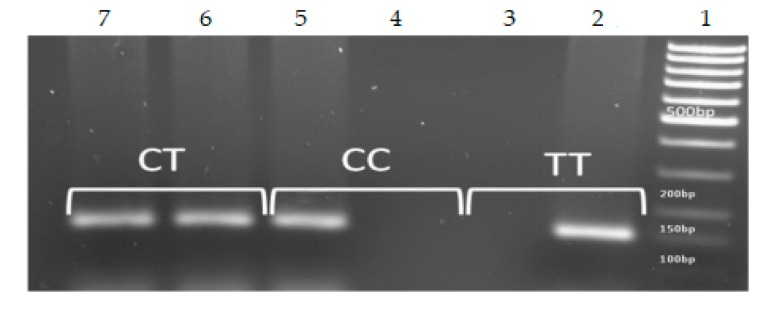
Polymorphism rs12979860—determination of genotypes (size of PCR product: 153 bp). Line 1, DNA ladder; lanes 2,3, TT homozygous; lanes 4,5, CC homozygous, Lines 6,7, CT heterozygous. Heterozygous samples resulted in the amplification of both bands, indicating the presence of the two alleles.

Polymorphism was evaluated using the Sequence-Specific Primer-Polymerase Chain Reaction (SSP-PCR) method, which multiplies a short DNA region upstream or downstream of the polymorphism with an allele-specific primer (Table 1) [21]. PCR products were analyzed in 2% agarose gel with ethidium bromide staining (Figure 1). Determination of individual-polymorphism genotypes was performed on the basis of the presence or absence of amplified target sequences. The amplification was performed using the following parameters: initial denaturation step at 95 °C for 5 min, then 35 cycles at 95 °C for 30 s, 58 °C for 45 s, 72 °C for 60 s, and 72 °C for 10 min.

### 2.5. Ethical Approval

This study was approved by the Ethics Committee and the Institutional Review Board of Clinical Center of Serbia, Belgrade (decision No. 57/14, approved on 21 March 2019). Please mention that the study was done according to the Helsinki declaration.

### 2.6. Statistical Analysis

The statistical analysis was performed with SPSS Statistics 20. We used the Chi-square test to determine if there was an association between *IL28b* genotypes and prevalence of HSV infections.

The Chi-square independence test was applied in order to determine a possible association between *IL28b* genotypes and incidence of HSK infection; *p*-values below 0.01 were considered statistically significant. The calculation of the total sample was based on the results of other authors who had studied the impact of the examined genotype in subjects with labial herpes and patients with hepatitis C. Using a G*Power 3.1.9.4 and χ2 test with a significance level (alpha) of 0.01 and a study strength of 0.08, it was deemed that a minimum number of 40 subjects was required to achieve significant results. The total sample size comprised 75 respondents; thus, the sample size was sufficient.

## 3. Results

### 3.1. Clinical Characteristics

This study included 75 participants with confirmed HSV IgG-positive status. Twenty-four patients had experienced recurrent HSK, and the remaining 51 individuals only had a history of recurrent HSL disease. The demographic characteristics of the study population are summarized in Table 2.

### 3.2. Distribution of IL28B Genotype Polymorphisms

**Figure 2:**
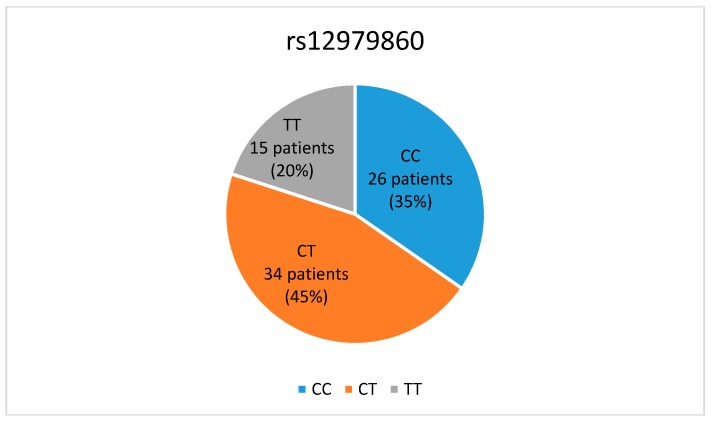
Distribution of the *IL28B* genotype rs12979860 in the study group as a whole. Percent distribution of the *IL28B* genotype rs12979860. CC: CCrs1279860, CT: CTrs1279860, TT: TTrs1279860.

The presence of the host *IL28B* rs1279860 genotype was investigated in all participants. The distribution of the polymorphism in the *IL28B* gene region for the study population as a whole is shown in Figure 2. CTrs1279860 was the most common genotype identified and was estimated to be present in 45% of patients (34 out of 75), followed by the CCrs1279860 genotype, present in 35%of patients (26 patients), and the TTrs1279860 genotype, observed in the remaining 20% of patients (15 patients) (Figure 2).

**Figure 3:**
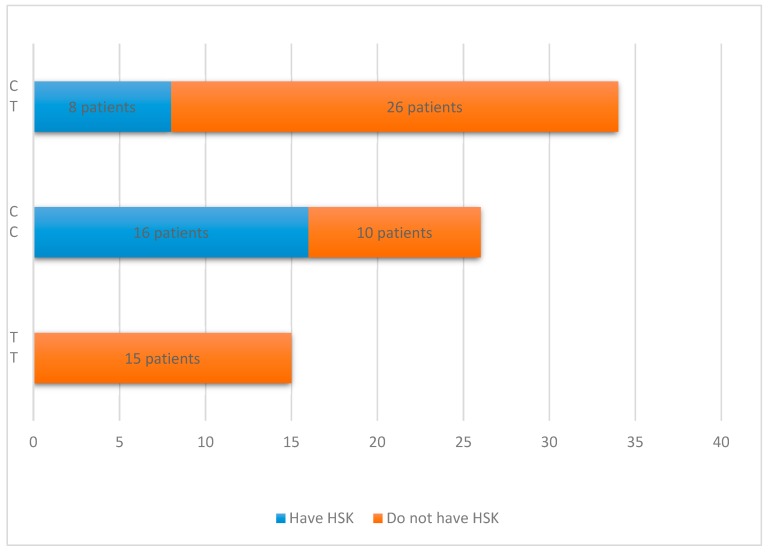
*IL28B* genotype distribution according to the clinical manifestation of recurrent herpes simplex virus (HSV) infection. Patients with herpes simplex keratitis (HSK) are marked in blue, and patients without HSK are marked in orange.

A significant association was observed between recurrent HSV keratitis and two SNPs of the *IL28B* genotype (CCrs12979860 and CTrs12979860, *p* < 0.01). Interestingly, the variation in CCrs12979860 showed a more significant association with recurrent HSV keratitis compared with CTrs12979860 (16 and 8 patients, respectively), as demonstrated in Figure 3. Furthermore, the TTrs12979860 genotype variant of *IL28B* was observed in 15 participants with HSV labialis disease. Of note, in our study, patients with recurrent HSV keratitis did not show the TTrs12979860 genotype variant (Figure 3).

## 4. Discussion

In our study, the polymorphism of the *IL28B* (rs12979860) gene was analyzed in HSV-1-seropositive patients with a history of recurrent HSV disease including HSV keratitis and/or herpes labialis. The CTrs12979860 genotype was the most common SNP variation in patients with recurrent HSV disease, followed by the CCrs12979860 and the TTrs12979860 genotypes. Interestingly, the majority of patients with recurrent HSV keratitis demonstrated the CC genotype (16 out of 24, 66.7%), whereas only 10 out of 51 (19.6%) individuals with herpes labialis (without a history of recurrent HSV eye disease) showed the CC genotype variant of *IL28B*. 

Recurrent stromal HSV keratitis is recognized as the most common infectious cause of vision-threatening corneal scarring in the developed world [22]. Accordingly, in our study, all patients with recurrent HSV keratitis exhibited severe corneal scarring accompanied by significant visual acuity reduction. The inflammatory process orchestrated by Th1 cells and non-lymphoid inflammatory cells leads to a chronic tissue-damaging response in addition to direct viral effects [4]. Most of the corneal damage results from neutrophil infiltration and neovascularization [23].

Studies with animal models have revealed that the genetic make-up of the host has an impact on the severity of HSV-1 corneal disease. Strains of inbred mice were shown to differ in their susceptibility to HSV corneal infection [24]. However, the roles of specific host genes in the resistance to HSK are poorly understood. Host genes may influence the outcome of an infection by affecting innate resistance factors as well as the function of the immune system [25]. It was also shown that susceptibility to HSV-1 infection is modulated by polymorphisms in genes that control the effector functions of cytotoxic T and natural killer (NK) lymphocytes [26]. 

A number of studies have investigated the relationship among host gene polymorphisms and both the severity and the recurrence of HSV infection [17,26,27]. In this context, it was recently reported that *IFN-λ* gene expression is associated with the recurrence and severity of recurrent HSV-1 disease [17]. Indeed, the minor T allele at rs12979860 was found to be associated with the severity and frequency of labial herpes recurrence. Griffiths et al. found that individuals with the CT or TT genotypes for *IL28B* had more frequent episodes of severe labial herpes, a condition resulting from the reactivation of HSV-1 [17]. On the basis of these findings, the authors suggested that recurrent HSV disease is likely to be associated with the *IL28B* polymorphism. Likewise, in our study, the CT and TT genotypes were more prevalent in individuals with isolated labial herpes. However, the CC genotype for *IL28B* was highly associated with recurrent HSK. It seems that both clinical manifestation and site of HSV reactivation from trigeminal ganglia might be related to different host genotypes as well as to the micro-environment. It is therefore of utmost clinical interest to identify a subgroup of patients with recurrent HSL who possess a higher risk for developing HSK, a potentially blinding disorder. Future studies with larger numbers of participants are necessary to confirm the hypothesis that the host genotype of *IL28B* might have an influence on determining which one of the three branches of trigeminal ganglion is most likely to be followed upon periodical HSV reactivation.

In patients with chronic hepatitis C, the CC genotype profile has been associated with a lower level of transcription of interferon-stimulated genes (ISGs) compared with TT individuals [28,29]. The non-CC genotypes, associated with a higher level of ISG expression in the setting of chronic hepatitis C infection, have previously been related to lower rates of spontaneous virus clearance. In contrast, the CC genotype, in which a low level ISG expression is observed, has been associated with high rates of spontaneous virus clearance [28].

The presence of the CC genotype variant in our patients with recurrent HSK might have led to incomplete control of infection and periodical viral shedding due to a lower transcription level of ISGs, as previously reported in patients with chronic hepatitis C infection [28,29]. 

Type III IFNs (IFN-λ) have been found to possess antiviral effects against HSV [30,31]. Pica et al. reported that reduced production of IFN-λ might be correlated with the development of recurrent HSV-1 labialis in immunocompetent individuals [32]. A recent study reported that IFN-λ treatment resulted in significant suppression of HSV-1 replication in both astrocytes and neurons. Furthermore, this was associated with the upregulation of endogenous IFN-α/β and several ISGs [13]. These findings indicate that IFN-λ functionally resembles that of type I IFN, inducing ISG expression and resulting in the suppression of viral replication [13]. Immunological control of virus reactivation should also be taken into account. The environmental and physiologic stimuli that induce HSV-1 reactivation from latency include exposure to UV light, stress, and immune suppression, suggesting a possible role for T cells in preventing viral reactivation [33].

## 5. Conclusions

In our study, the CTrs12979860 genotype was found to be the most common SNP variation in patients with recurrent HSV disease, followed by CCrs12979860 and TTrs12979860.

While the CT and TT genotypes were more prevalent in individuals with isolated labial herpes, our results indicate that HSV-1-seropositive individuals expressing the CC *IL28B* genotype have a tendency toward developing recurrent herpetic keratitis. Clinical manifestation and the severity of recurrent HSV-1 infection seem to be influenced by polymorphism of the *IL28B* genotype. Further studies on host genetics in patients with recurrent herpetic eye disease are necessary to support our results.

## Figures and Tables

**Table 1:** Nucleotide sequences of the primers used in this study. SNP: single-nucleotide polymorphism.

*IL28B* SNP	Nucleotide Sequence	Size of PCR Product
rs12979860	Gen (sense) 5′-TTATCGCATACGG CTAGGC-3′	153 bp
	C (antisense) 5′TGCAATTCAACCCTGGTTC G-3′	
	T (antisense) 5′ TGCAATTCAACCCTGGTTC A-3	

**Table 2:** Demographic characteristics of the study population.

*Characteristics*	*All Patients*
n	75
Gender (M/F)	36:39
Age, y	44.95 ± 11.294
Have keratitis (M)	5 (6.7%)
Do not have keratitis (M)	31 (41.3%)
Have keratitis (F)	19 (25.3%)
Do not have keratitis (F)	20 (26.7%)

Data are reported as mean ± standard deviation. M, male; F, female.
